# A case report of scrub typhus complicated with myocarditis and rhabdomyolysis

**DOI:** 10.1186/s12879-018-3458-1

**Published:** 2018-11-07

**Authors:** Young-Jae Ki, Dong-Min Kim, Na-Ra Yoon, Sung-Soo Kim, Choon-Mee Kim

**Affiliations:** 1 Departments of Internal Medicine, College of Medicine, Chosun University, 588 Seosuk-dong, Dong-gu, Gwangju, 61453 Republic of Korea; 2 Premedical Science, College of Medicine, Chosun University, Gwangju, Republic of Korea

**Keywords:** Scrub typhus, Myocarditis, Rhabdomyolysis

## Abstract

**Background:**

Scrub typhus is a zoonotic disease caused by *Orientia tsutsugamushi*, a gram-negative intracellular bacterium. Myocarditis and rhabdomyolysis are rare complications of scrub typhus.

**Case presentation:**

We report a case of scrub typhus, which was simultaneously complicated with myocarditis and rhabdomyolysis. A 54-year-old woman presented to our hospital with myalgia in the upper and lower limbs, oedema and a fever of 7 days’ duration. We confirmed the diagnosis of scrub typhus complicated with myocarditis by pericardial fluid analysis and cardiac magnetic resonance imaging results. The pericardial fluid showed characteristics of an exudate, an elevated immunofluorescence assay (IFA) IgG titer of 1:2048 and a positive 16S rRNA qPCR result. We also diagnosed rhabdomyolysis by the patient’s presenting symptoms, elevated muscle enzyme levels and bone scan results.

**Conclusion:**

We report for the first time a case of scrub typhus complicated with both myocarditis and rhabdomyolysis, the causative agent of which was the Boryong genotype of *O. tsutsugamushi*.

## Background

Scrub typhus is a mite-borne infectious disease caused by *Orientia tsutsugamushi*, a gram-negative intracellular bacterium. This infection is prevalent in rural East Asia and the Western Pacific islands. It is usually characterized by the acute onset of fever, chills, rash, and eschar, and patients can be easily managed by early diagnosis and treatment with doxycycline or tetracycline. However, severe complications such as acute respiratory distress syndrome, meningoencephalitis, pneumonitis, acute renal failure and myocarditis infrequently occur and can be fatal.

To date, cardiac involvement (e.g., myocarditis, pericarditis, and infective endocarditis) or rhabdomyolysis in scrub typhus has been intermittently reported [1–6]. Furthermore, the simultaneous complication of scrub typhus with both myocarditis and rhabdomyolysis has not been reported. Here, we report a case of scrub typhus in a 54-year-old woman presenting with myocarditis and rhabdomyolysis.

## Case presentation

A 54-year-old woman with no previous comorbidity was brought to our Emergency Department for further evaluation of increased levels of muscle enzymes and cardiac enzymes. Prior to admission, she was admitted in a local clinic with myalgia in the upper and lower limbs, oedema and a fever of 7 days’ duration. She was diagnosed clinically with scrub typhus by the presence of an eschar in the area of the right shin and was treated with 100 mg doxycycline for 2 days. The occupation of patient was housewife. Upon our physical examination, the blood pressure was 120/80 mmHg, the pulse rate was 101 beats/min, the respiratory rate was 18 breaths/min, and the body temperature was 36.7 °C. She was alert and fully oriented. Auscultation of both lungs revealed mild rales in both lower lobes. No heart murmur was audible. The eschar was observed in the area of the right shin.

**Fig. 1:**
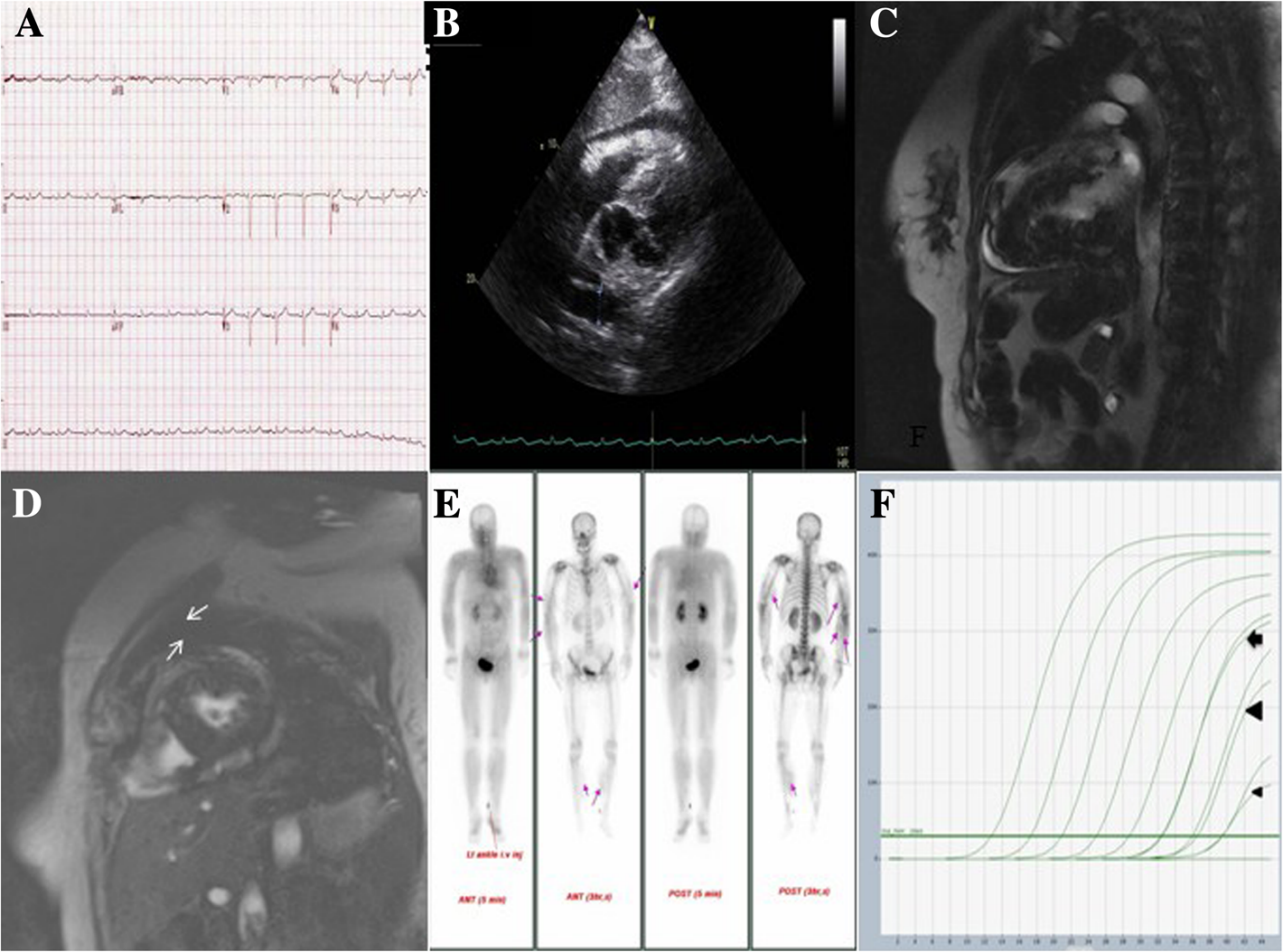
The electrocardiogram (ECG) performed upon admission showed a normal sinus rhythm with a low QRS voltage in all limb leads and precordial leads (**a**). The TTE subcostal view revealed a normally sized left ventricle and a mild pericardial effusion (**b**). Contrast-enhanced cardiac magnetic resonance imaging showed a pericardial effusion and subepicardial enhancement in the apical segment (**c**). Contrast-enhanced cardiac magnetic resonance imaging showed subepicardial enhancement in the mid anteroseptal and mid anterior segments (**d**). A bone scan revealed increased soft tissue uptake in both the arms and the legs (**e**). Real-time PCR targeting the 16S rRNA gene of *O. tsutsugamushi* showed positive at a cross point cycle (Cp) of 32.3 (arrow) in a pericardial fluid specimen, 35.97 (large arrow head) in an eschar specimen, and 39.42 (small arrow head) in an endocardial tissue specimen (positive control: Karp genotype, Cp value = 19.71) (**f**)

An electrocardiogram (ECG) performed in the emergency room showed a normal sinus rhythm with a low QRS voltage in all limb leads and precordial leads (Fig. 1a). Chest X-ray revealed a slightly increased cardiothoracic ratio. Laboratory testing showed elevation of the following parameters: white blood cell count (15,980/μL, normal = 4000–10,800/μL), erythrocyte sedimentation rate (35 mm/hr., normal range = 0–30 mm/hr), C-reactive protein (2.13 mg/dL, normal = 0–0.3 mg/dL), aspartate aminotransferase (75.9 IU/L, normal = 10–40 IU/L), creatine phosphokinase (CPK) (3337 U/L, normal = 55–215 U/L), creatinine kinase-myocardial band (CK-MB) (104.6 ng/mL, normal = 0–4.88 ng/mL), troponin I (0.055 ng/mL, normal = 0–0.016 ng/mL), myoglobin (2498 ng/mL, normal = 25–58 ng/mL), prohormone of brain natriuretic peptide (proBNP) (477.6 pg/mL, normal = 0–270 pg/mL) and blood urea nitrogen (36.4 mg/dL, normal = 8.0–20 mg/dL). Additionally, laboratory testing showed normal levels of creatine (0.66 mg/dL, normal = 0.5–1.3 mg/dL) and potassium (4.4 mEq/L, normal = 3.5–5.0 mEq/L) and a decreased level of albumin (3.07 g/dL, normal = 3.5–5.2 g/dL). Transthoracic echocardiography (TTE) revealed normal left ventricular systolic function with an ejection fraction of 62%, along with mild pericardial effusion (Fig. 1b).

First of all, scrub typhus with rhabdomyolysis was suspected, administration of intravenous fluid and doxycycline (200 mg/day) was initiated immediately. We confirmed that the serum indirect immunofluorescence assay (IFA) IgM titer against *O. tsutsugamushi* was < 1:16 and that the IgG titer was 1:4096. In addition, the nested polymerase chain reaction (PCR) targeting the *O. tsutsugamushi* 56-kDa protein-encoding gene was negative in a specimen from the blood buffy coat, but positive in an eschar specimen. A comparative analysis of the *O. tsutsugamushi* DNA sequence obtained from the eschar with sequences in the GenBank database confirmed that the patient was infected with the Boryong genotype [7]. PCR tests to detect Hantavirus, severe fever thrombocytopenia syndrome virus, and species of Anaplasma, Ehrlichia, and Borrelia were all negative [8–11] (Table 1).

**Table 1:** Scrub typhus polymerase chain reaction and immunofluorescence assay results by sample collection date

Sample collection date	Sample name	Scrub typhus PCR			Scrub typhus IFA		HFRS	SFTS	Anaplasma	Borrelia
		56 kDa kit	16S qPCR		IgM	IgG	(RT-N PCR)	RT-PCR	(groEL)	
2016-11-28^a^	buffy & plasma	Negative		Negative	< 1:16	1:4096	Negative	Negative	Negative	Negative
	eschar	Positive (Boryong)		Positive					Negative	
2016-12-02	pericardial fluid	Negative		Positive	< 1:16	1:2048	Negative		Negative	
	tissue (cardiac)	Negative		Positive					Negative	
2016-12-05	buffy & plasma				< 1:16	1:2048				
2016-12-14	buffy & plasma				< 1:16	1:2048				

^a^Antibiotics (doxycycline) had been given previously in a local clinic for two days and additionally given in our hospital for six days beginning on 2016-11-28

During the patient’s hospitalization, muscle enzyme and cardiac enzyme levels increased continuously. On day 3 of hospitalization, the creatine phosphokinase level was 18,262 U/L (normal = 55–215 U/L), the CK-MB level was 272.3 ng/mL (normal = 0–4.88 ng/mL), the troponin I level was 1.62 ng/mL (normal = 0–0.016 ng/mL) and the myoglobin level peaked at 3000 ng/mL (normal = 25–58 ng/mL). Despite the lack of specific symptoms, we suspected myocarditis based on the ECG results, TTE imaging findings and the rapid increase in cardiac enzyme levels. Therefore, cardiac magnetic resonance imaging (MRI) was performed. The cardiac MRI demonstrated normal left ventricular function (58.9%) with a large amount of pericardial effusion. The delayed enhancement images revealed a subepicardial enhancement involving the basal lateral, mid anteroseptal, mid anterior and apical segments of the left ventricle wall (Fig. 1c, d).

On day 5 of hospitalization, we performed pericardiocentesis due to the large amount of pericardial effusion without concomitant tamponade physiology, and 195 cc of serous pericardial fluid was aspirated. The pericardial fluid and buffy coat of the patient was inoculated onto L929 cells to isolate the causative microorganisms, but no microorganisms could be isolated. Real-time PCR targeting the *O. tsutsugamushi* 16S rRNA gene using a pericardial fluid specimen showed a positive result at a crossing point cycle (Cp) of 32.3, and qPCR using an eschar specimen was positive at a Cp of 35.97 [12] (Fig. 1f). The pericardial fluid analysis showed a white blood cell count of 150/mm^3^ (80% monocytes), a total protein level of 4.08 g/dL, a fluid/serum protein ratio of 0.77, a lactate dehydrogenase (LDH) level of 764 U/L, and a fluid/serum LDH ratio of 0.65. By these results, the pericardial fluid was classified as an exudate [13]. The adenosine deaminase level was 21.7 U/L (normal = 5.8–23 U/L), the bacterial and fungal cultures were sterile, and the IFA IgM titer against *O. tsutsugamushi* was < 1:16 but the IgG titer was 1:2048 in the pericardial fluid. On the same day, coronary angiography for a differential diagnosis of myocardial infarction revealed no abnormalities. Based on the cardiac MRI results, we performed endomyocardial biopsy (EMB) to evaluate a definite diagnosis of myocarditis. The biopsy specimen consisted of five pieces, which was barely sufficient for real-time PCR, but the pathology report indicated that the specimens contained inadequate tissue for definitive diagnosis. However, we could confirm the diagnosis of scrub typhus myocarditis based on the elevated cardiac enzymes, the pericardial fluid analysis results, and the TTE and cardiac MRI imaging findings. On day of 8 of hospitalization, a follow-up TTE revealed normal left ventricular function with no pericardial effusion.

On day 10 of hospitalization, we also confirmed the diagnosis of rhabdomyolysis from the bone scan, which revealed increased soft tissue uptake in both arms and legs (Fig. 1e). The patient was given continuous intravenous fluid and diuretics for the management of rhabdomyolysis, a 6-day course of doxycycline for the scrub typhus infection and conservative therapy for myocarditis. The patient’s renal function and potassium level remained within the normal range throughout the hospitalization. The cardiac enzyme and muscle enzyme levels decreased. On day 16 of hospitalization, the CPK level had decreased to 595 U/L (normal = 55–215 U/L), the CK-MB level was within the normal range at 4.140 ng/mL (normal = 0–4.88 ng/mL) and the troponin I level had decreased to 0.096 ng/mL (normal = 0–0.016 ng/mL). The patient was discharged on day 17 of hospitalization after resolution of her presenting symptoms.

## Discussion and conclusions

Myocarditis can present with a wide range of clinical manifestations, from nonspecific symptoms such as fever, myalgia, palpitation and exertional dyspnea to cardiogenic shock or sudden cardiac death [14]. As in our case, the clinical presentation of myocarditis can be deceptive due to the absence of symptoms, and myocarditis should be considered in cases of systemic infection with concomitant new cardiovascular dysfunctions or elevated cardiac enzymes. Myocarditis also mimics myocardial infarction clinically; therefore, coronary artery disease should be included in the differential diagnosis for myocarditis. Viral infections are known to be the most common cause of myocarditis, and many cases of myocarditis caused by the varicella zoster virus, the human immunodeficiency virus and coxsackievirus have been reported [15, 16]. In comparison, bacterial myocarditis is relatively uncommon [17]. *O. tsutsugamushi* is primarily localized in the endothelial cells of the heart, lung, brain, kidney, and skin; and within cardiac muscle cells [18]. Subsequently, infection with *O. tsutsugamushi* results in vasculitis in multiple organs, leading to various complications. Among these complications, cardiac manifestations such as myocarditis, pericarditis and infective endocarditis have been reported [1, 2, 4–6].

EMB results are essential in confirming the diagnosis of myocarditis, but this technique is invasive in haemodynamically unstable patients and also lacks sensitivity [14]. Practically, EMB were used to diagnose myocarditis in 111 of 1230 patients (9%) with unexplained cardiomyopathy in one large study [19]. In addition, only one case of scrub typhus myocarditis has been confirmed by EMB in Japan [20]. In the present case, EMB was performed but we obtained an inadequate specimen amount for definitive diagnosis. However, many researchers have noted strong clinical, ventriculographic, and laboratory evidence of myocarditis among patients with negative biopsies [21, 22]. Recently, cardiac MRI is increasingly being used for the diagnosis and prognostic assessment of myocarditis. In addition, cardiac MRI has three advantages that make it the leading tool for diagnosing myocarditis. First, cardiac MRI enables clinicians to assess cardiac function indirectly. Second, it provides assistance in directing myocardial biopsy to appropriate locations. In comparison with off-target sampling, myocardial biopsy yields improve substantially when combined with cardiac MRI if the sampling is performed in focal regions detected on the cardiac MRI. Lastly, cardiac MRI helps clinicians to differentiate myocarditis from myocardial infarction by the myocardial delayed enhancement pattern [23].

In our case, the patient was administered antibiotics before we obtained her blood sample; therefore, the nested PCR was negative using the buffy coat, but we could confirm scrub typhus by a positive PCR assay using a specimen from the eschar, which is an inoculation site of *O. tsutsugamushi* that presumably contains a high inoculum density. We could also diagnose scrub typhus myocarditis by elevated cardiac biomarker (creatine kinase-MB, cardiac troponin T, and proBNP) levels; positive PCR; elevated IFA IgG titer in pericardial fluid; and noninvasive test results such as ECG, echocardiography and cardiac MRI.

To our knowledge, this is the first case of scrub typhus myocarditis with the Boryong genotype genetically confirmed as the causative agent. In addition, we performed pericardial fluid analysis for the first time in a scrub typhus patient with myocarditis or pericarditis. The pericardial effusion had characteristics of an exudate and was PCR-positive for *O. tsutsugamushi* 16S rRNA. Taken together, we can assume that scrub typhus myocarditis occurred by direct invasion of *O. tsutsugamushi* [12]. Further study is needed to elucidate the mechanisms contributing to myocarditis due to *O. tsutsugamushi*.

Rhabdomyolysis is caused by tissue hypoxia, metabolic abnormality, toxin release and bacterial invasion. Rhabdomyolysis caused by bacterial infection has been reported occasionally but there have been only 2 case reports about rhabdomyolysis associated with scrub typhus [3, 24]. Clinical manifestations of rhabdomyolysis present as myalgia, general weakness, dark-coloured urine and acute renal failure. Our patient presented with myalgia in both upper and lower limbs and elevated levels of serum CPK and myoglobin, and the diagnosis was ultimately confirmed from a bone scan. The proposed mechanism leading to rhabdomyolysis in scrub typhus is the same as for myocarditis and other vasculitic complications. Cardiac enzyme levels are a very useful tool for diagnosing myocardial injury but can be falsely elevated in rhabdomyolysis.

The treatment strategy for rhabdomyolysis and myocarditis is different; for example, intravenous hydration is beneficial for prevention of acute kidney injury in rhabdomyolysis but can be harmful in myocarditis with depressed left ventricular function. Although myocarditis and rhabdomyolysis are rare complications of scrub typhus individually, both complications can occur together, as in our case. Therefore, a careful workup should be considered for scrub typhus patients with elevated cardiac enzymes to determine whether the elevated levels originate from ischaemic conditions or myocarditis or are falsely elevated due to rhabdomyolysis.

Our case report includes two limitations. First, no microorganism was cultured from either the pericardial fluid or the buffy coat, possibly due to the early administration of antibiotics. Second, although we performed EMB, we obtained an inadequate sample amount for definitive diagnosis of myocarditis.

Here, we reported a case of scrub typhus caused by the Boryong genotype of *O. tsutsugamushi*, which was complicated with both myocarditis and rhabdomyolysis for the first time. Analysis of the pericardial fluid showed that the pericardial effusion had characteristics of an exudate, a high IgG IFA antibody titer and a positive qPCR result for *O. tsutsugamushi* 16S rRNA.

## Abbreviations

CK-MB: Creatinine kinase-myocardial band
Cp: Crossing points
CPK: Creatine phosphate kinase
ECG: Electrocardiogram
EMB: Endomyocardial biopsy
IFA: Immunofluorescence assay
LDH: Lactate dehydrogenase
MRI: Magnetic resonance imaging
PCR: Polymerase chain reaction
ProBNP: Prohormone of brain natriuretic peptide
TTE: Transthoracic echocardiography
